# Supplementary material for: Investigation of the Effects of Metallic Nanoparticles on Fertility Outcomes and Endocrine Modification of the Hypothalamic-Pituitary-Gonadal Axis
Source: Int J Mol Sci. 2023 Jul 20;24(14):11687. doi: 10.3390/ijms241411687 (PMC10380468; doi:10.3390/ijms241411687)
Supplement: Supplementary file 1 [file ijms-24-11687-s001.zip › ijms-2451956-supplementary.pdf]

Investigation of the Effects of Metallic Nanoparticles on Fertility Outcomes and Endocrine Modification of the Hypothalamic-Pituitary-Gonadal Axis

Miguel A. Sogorb, Héctor Candela, Jorge Estévez and Eugenio Vilanova

SUPPELEMNTARY MATERIAL INDEX

Supplementary Table S1…………………………………………………………………...……1

Supplementary Figure S1………………………………………………………………..……7

Supplementary Table S1: List of the 237 genes included in the GnRH receptor pathway according to PANTHER.

Gene ID (Uniprot)	Gene Name
P60953	Cell division control protein 42 homolog
P37231	Peroxisome proliferator-activated receptor gamma
P22736	Nuclear receptor subfamily 4 group A member 1
P08476	Inhibin beta A chain
Q96A54	Adiponectin receptor protein 1
Q8IVT5	Kinase suppressor of Ras 1
P01229	Lutropin subunit beta
P45983	Mitogen-activated protein kinase 8
P55347	Homeobox protein PKNOX1
P04150	Glucocorticoid receptor
P29475	Nitric oxide synthase, brain
Q9NQ66	1-phosphatidylinositol 4,5-bisphosphate phosphodiesterase beta-1
Q05397	Focal adhesion kinase 1
Q9UP65	Cytosolic phospholipase A2 gamma
P54619	5'-AMP-activated protein kinase subunit gamma-1
Q16539	Mitogen-activated protein kinase 14
P16050	Polyunsaturated fatty acid lipoxygenase ALOX15
Q13705	Activin receptor type-2B
P28562	Dual specificity protein phosphatase 1
P80108	Phosphatidylinositol-glycan-specific phospholipase D
Q13485	Mothers against decapentaplegic homolog 4
Q9NZJ5	Eukaryotic translation initiation factor 2-alpha kinase 3
Q99683	Mitogen-activated protein kinase kinase kinase 5
O43318	Mitogen-activated protein kinase kinase kinase 7
P01308	Insulin
P16989	Y-box-binding protein 3
Q9BY44	Eukaryotic translation initiation factor 2A
P17252	Protein kinase C alpha type
P28482	Mitogen-activated protein kinase 1

P52564	Dual specificity mitogen-activated protein kinase kinase 6
P18146	Early growth response protein 1
Q14643	Inositol 1,4,5-trisphosphate receptor type 1
Q02779	Mitogen-activated protein kinase kinase kinase 10
Q6UUV9	CREB-regulated transcription coactivator 1
P31321	cAMP-dependent protein kinase type I-beta regulatory subunit
P36402	Transcription factor 7
Q9HCS4	Transcription factor 7-like 1
Q07889	Son of sevenless homolog 1
P08758	Annexin A5
O75343	Guanylate cyclase soluble subunit beta-2
P62993	Growth factor receptor-bound protein 2
Q9Y6R4	Mitogen-activated protein kinase kinase kinase 4
P34995	Prostaglandin E2 receptor EP1 subtype
P01100	Proto-oncogene c-Fos
P18509	Pituitary adenylate cyclase-activating polypeptide
O14775	Guanine nucleotide-binding protein subunit beta-5
P50281	Matrix metalloproteinase-14
Q9HCQ7	Pro-FMRFamide-related neuropeptide V
P31749	RAC-alpha serine/threonine-protein kinase
P35568	Insulin receptor substrate 1
Q02535	DNA-binding protein inhibitor ID-3
P16520	Guanine nucleotide-binding protein G(I)/G(S)/G(T) subunit beta-3
Q08828	Adenylate cyclase type 1
Q15121	Astrocytic phosphoprotein PEA-15
P30968	Gonadotropin-releasing hormone receptor
P14416	D(2) dopamine receptor
O43741	5'-AMP-activated protein kinase subunit beta-2
P43116	Prostaglandin E2 receptor EP2 subtype
P01225	Follitropin subunit beta
Q13554	Calcium/calmodulin-dependent protein kinase type II subunit beta
Q13506	NGFI-A-binding protein 1
P43694	Transcription factor GATA-4
Q01668	Voltage-dependent L-type calcium channel subunit alpha-1D
P17275	Transcription factor jun-B
P63096	Guanine nucleotide-binding protein G(i) subunit alpha-1
Q13936	Voltage-dependent L-type calcium channel subunit alpha-1C
P19419	ETS domain-containing protein Elk-1
P49023	Paxillin
Q07869	Peroxisome proliferator-activated receptor alpha
O95644	Nuclear factor of activated T-cells, cytoplasmic 1
P36896	Activin receptor type-1B
Q9HAV0	Guanine nucleotide-binding protein subunit beta-4
P53778	Mitogen-activated protein kinase 12
O95819	Mitogen-activated protein kinase kinase kinase kinase 4
P08069	Insulin-like growth factor 1 receptor
O95972	Bone morphogenetic protein 15
P17535	Transcription factor jun-D

Q9BQE3	Tubulin alpha-1C chain
P23511	Nuclear transcription factor Y subunit alpha
Q14573	Inositol 1,4,5-trisphosphate receptor type 3
P23582	C-type natriuretic peptide
P00403	Cytochrome c oxidase subunit 2
P0DMV8	Heat shock 70 kDa protein 1A
O14654	Insulin receptor substrate 4
P59768	Guanine nucleotide-binding protein G(I)/G(S)/G(O) subunit gamma-2
Q15759	Mitogen-activated protein kinase 11
P18075	Bone morphogenetic protein 7
P04049	RAF proto-oncogene serine/threonine-protein kinase
Q9UGJ0	5'-AMP-activated protein kinase subunit gamma-2
Q07890	Son of sevenless homolog 2
P01236	Prolactin
Q13233	Mitogen-activated protein kinase kinase kinase 1
O60840	Voltage-dependent L-type calcium channel subunit alpha-1F
Q05655	Protein kinase C delta type
P13521	Secretogranin-2
P03971	Muellerian-inhibiting factor
P36894	Bone morphogenetic protein receptor type-1A
Q02363	DNA-binding protein inhibitor ID-2
Q99697	Pituitary homeobox 2
Q86U70	LIM domain-binding protein 1
O15198	Mothers against decapentaplegic homolog 9
P61371	Insulin gene enhancer protein ISL-1
Q14289	Protein-tyrosine kinase 2-beta
P06401	Progesterone receptor
P49841	Glycogen synthase kinase-3 beta
P05771	Protein kinase C beta type
P50553	Achaete-scute homolog 1
Q15742	NGFI-A-binding protein 2
P48539	Calmodulin regulator protein PCP4
Q8IVH8	Mitogen-activated protein kinase kinase kinase kinase 3
Q04206	Transcription factor p65
P29992	Guanine nucleotide-binding protein subunit alpha-11
Q92831	Histone acetyltransferase KAT2B
P01137	Transforming growth factor beta-1 proprotein
P56199	Integrin alpha-1
P09529	Inhibin beta B chain
P63000	Ras-related C3 botulinum toxin substrate 1
P68363	Tubulin alpha-1B chain
Q13285	Steroidogenic factor 1
P20594	Atrial natriuretic peptide receptor 2
P40424	Pre-B-cell leukemia transcription factor 1
Q04759	Protein kinase C theta type
Q13574	Diacylglycerol kinase zeta
P22004	Bone morphogenetic protein 6
Q12968	Nuclear factor of activated T-cells, cytoplasmic 3

P04899	Guanine nucleotide-binding protein G(i) subunit alpha-2
Q86V24	Adiponectin receptor protein 2
P12931	Proto-oncogene tyrosine-protein kinase Src
Q9BRK5	45 kDa calcium-binding protein
Q9UHY1	Nuclear receptor-binding protein
Q05513	Protein kinase C zeta type
P27037	Activin receptor type-2A
Q12852	Mitogen-activated protein kinase kinase kinase 12
Q92918	Mitogen-activated protein kinase kinase kinase kinase 1
A7KAX9	Rho GTPase-activating protein 32
P16471	Prolactin receptor
P37275	Zinc finger E-box-binding homeobox 1
P18847	Cyclic AMP-dependent transcription factor ATF-3
Q99558	Mitogen-activated protein kinase kinase kinase 14
P15336	Cyclic AMP-dependent transcription factor ATF-2
Q13698	Voltage-dependent L-type calcium channel subunit alpha-1S
Q99759	Mitogen-activated protein kinase kinase kinase 3
P61224	Ras-related protein Rap-1b
P00533	Epidermal growth factor receptor
Q13873	Bone morphogenetic protein receptor type-2
Q08209	Serine/threonine-protein phosphatase 2B catalytic subunit alpha isoform
P0DMV9	Heat shock 70 kDa protein 1B
P27986	Phosphatidylinositol 3-kinase regulatory subunit alpha
P40763	Signal transducer and activator of transcription 3
P11166	Solute carrier family 2, facilitated glucose transporter member 1
Q03135	Caveolin-1
P41279	Mitogen-activated protein kinase kinase kinase 8
Q9Y3S1	Serine/threonine-protein kinase WNK2
O43283	Mitogen-activated protein kinase kinase kinase 13
Q9H2B2	Synaptotagmin-4
P27361	Mitogen-activated protein kinase 3
O15264	Mitogen-activated protein kinase 13
Q14571	Inositol 1,4,5-trisphosphate receptor type 2
O00238	Bone morphogenetic protein receptor type-1B
P32242	Homeobox protein OTX1
Q71U36	Tubulin alpha-1A chain
Q05193	Dynamin-1
P46734	Dual specificity mitogen-activated protein kinase kinase 3
P45984	Mitogen-activated protein kinase 9
P14859	POU domain, class 2, transcription factor 1
A6NIZ1	Ras-related protein Rap-1b-like protein
P12643	Bone morphogenetic protein 2
P08047	Transcription factor Sp1
P09471	Guanine nucleotide-binding protein G(o) subunit alpha
P01112	GTPase Hras
Q15797	Mothers against decapentaplegic homolog 1
P01133	Pro-epidermal growth factor

Q09472	Histone acetyltransferase p300
Q02750	Dual specificity mitogen-activated protein kinase kinase 1
Q9Y4H2	Insulin receptor substrate 2
Q9NSY0	Nuclear receptor-binding protein 2
P78337	Pituitary homeobox 1
P47712	Cytosolic phospholipase A2
P01148	Progonadoliberin-1
P43088	Prostaglandin F2-alpha receptor
P05412	Transcription factor AP-1
P05111	Inhibin alpha chain
P05556	Integrin beta-1
Q13469	Nuclear factor of activated T-cells, cytoplasmic 2
Q9BYP7	Serine/threonine-protein kinase WNK3
P16220	Cyclic AMP-responsive element-binding protein 1
P53539	Protein fosB
P12644	Bone morphogenetic protein 4
Q99717	Mothers against decapentaplegic homolog 5
Q16584	Mitogen-activated protein kinase kinase kinase 11
O14733	Dual specificity mitogen-activated protein kinase kinase 7
Q9Y6Q9	Nuclear receptor coactivator 3
Q9Y4K4	Mitogen-activated protein kinase kinase kinase kinase 5
P46098	5-hydroxytryptamine receptor 3A
P35222	Catenin beta-1
P36507	Dual specificity mitogen-activated protein kinase kinase 2
Q03167	Transforming growth factor beta receptor type 3
P08754	Guanine nucleotide-binding protein G(i) subunit alpha-3
Q15583	Homeobox protein TGIF1
P19883	Follistatin
P80192	Mitogen-activated protein kinase kinase kinase 9
Q15796	Mothers against decapentaplegic homolog 2
Q02153	Guanylate cyclase soluble subunit beta-1
Q9Y2U5	Mitogen-activated protein kinase kinase kinase 2
O15534	Period circadian protein homolog 1
Q5JWF2	Guanine nucleotide-binding protein G(s) subunit alpha isoforms XLas
Q12851	Mitogen-activated protein kinase kinase kinase kinase 2
P11831	Serum response factor
P63162	Small nuclear ribonucleoprotein-associated protein N
O60733	85/88 kDa calcium-independent phospholipase A2
Q02156	Protein kinase C epsilon type
P35408	Prostaglandin E2 receptor EP4 subtype
P84022	Mothers against decapentaplegic homolog 3
P41586	Pituitary adenylate cyclase-activating polypeptide type I receptor
P12757	Ski-like protein
P62873	Guanine nucleotide-binding protein G(I)/G(S)/G(T) subunit beta-1
Q9H4A3	Serine/threonine-protein kinase WNK1
Q99811	Paired mesoderm homeobox protein 2
P50148	Guanine nucleotide-binding protein G(q) subunit alpha
P05019	Insulin-like growth factor I

P62879	Guanine nucleotide-binding protein G(I)/G(S)/G(T) subunit beta-2
Q13131	5'-AMP-activated protein kinase catalytic subunit alpha-1
P18206	Vinculin
Q16671	Anti-Muellerian hormone type-2 receptor
P11474	Steroid hormone receptor ERR1
P61812	Transforming growth factor beta-2 proprotein
P06213	Insulin receptor
P50458	LIM/homeobox protein Lhx2
P23769	Endothelial transcription factor GATA-2
P43115	Prostaglandin E2 receptor EP3 subtype
P43119	Prostacyclin receptor
Q92793	CREB-binding protein
P10275	Androgen receptor
P01215	Glycoprotein hormones alpha chain
Q9Y478	5'-AMP-activated protein kinase subunit beta-1
Q15848	Adiponectin
Q14934	Nuclear factor of activated T-cells, cytoplasmic 4

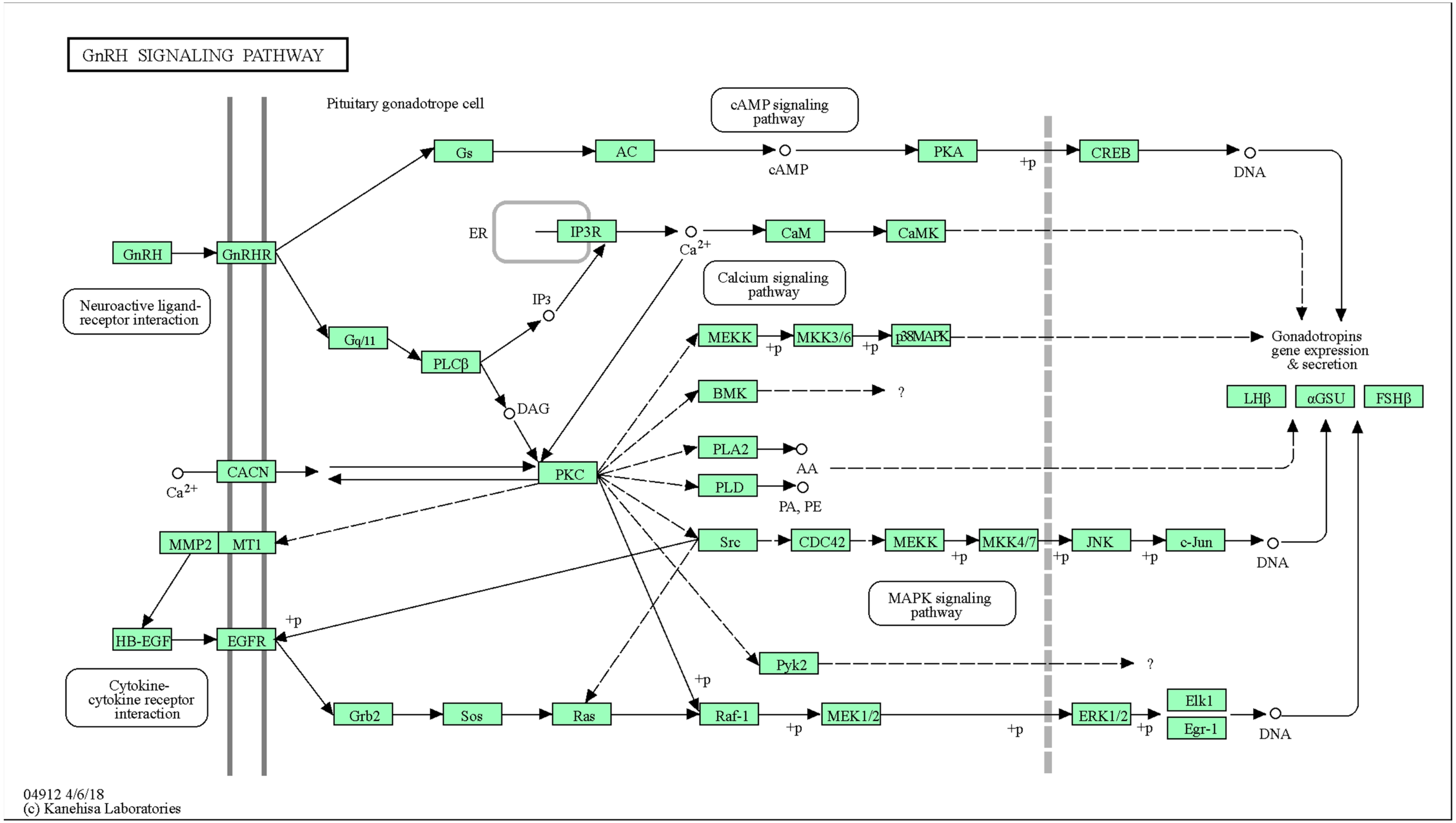

Figure S1. GnRH signaling pathway for *Homo sapiens*. Provided by KEGG (hsa04912). Reproduced with permission.
